# *SCN1A* Mutation—Beyond Dravet Syndrome: A Systematic Review and Narrative Synthesis

**DOI:** 10.3389/fneur.2021.743726

**Published:** 2021-12-24

**Authors:** Jiangwei Ding, Xinxiao Li, Haiyan Tian, Lei Wang, Baorui Guo, Yangyang Wang, Wenchao Li, Feng Wang, Tao Sun

**Affiliations:** ^1^Department of Neurosurgery, General Hospital of Ningxia Medical University, Yinchuan, China; ^2^Ningxia Key Laboratory of Cerebrocranial Disease, The Incubation Base of National Key Laboratory, Ningxia Medical University, Yinchuan, China; ^3^Department of Neurosurgery, The Fifth Affiliated Hospital of Zhengzhou University, Zhengzhou, China; ^4^Department of Neurology, First Affiliated Hospital of Zhengzhou University, Zhengzhou, China; ^5^Department of Neurosurgery, The First Affiliated Hospital of Xinxiang Medical University, Weihui, China; ^6^Department of Neurosurgery, The First Affiliated Hospital of Zhejiang University School of Medicine, Hangzhou, China

**Keywords:** SCN1A, Dravet syndrome, GEFS+, migraine, autism spectrum disorder

## Abstract

**Background:** *SCN1A* is one of the most common epilepsy genes. About 80% of *SCN1A* gene mutations cause Dravet syndrome (DS), which is a severe and catastrophic epileptic encephalopathy. More than 1,800 mutations have been identified in *SCN1A*. Although it is known that *SCN1A* is the main cause of DS and genetic epilepsy with febrile seizures plus (GEFS+), there is a dearth of information on the other related diseases caused by mutations of *SCN1A*.

**Objective:** The aim of this study is to systematically review the literature associated with *SCN1A* and other non-DS-related disorders.

**Methods:** We searched PubMed and SCOPUS for all the published cases related to gene mutations of *SCN1A* until October 20, 2021. The results reported by each study were summarized narratively.

**Results:** The PubMed and SCOPUS search yielded 2,889 items. A total of 453 studies published between 2005 and 2020 met the final inclusion criteria. Overall, 303 studies on DS, 93 on GEFS+, three on Doose syndrome, nine on the epilepsy of infancy with migrating focal seizures (EIMFS), six on the West syndrome, two on the Lennox–Gastaut syndrome (LGS), one on the Rett syndrome, seven on the nonsyndromic epileptic encephalopathy (NEE), 19 on hemiplegia migraine, six on autism spectrum disorder (ASD), two on nonepileptic *SCN1A*-related sudden deaths, and two on the arthrogryposis multiplex congenital were included.

**Conclusion:** Aside from DS, *SCN1A* also causes other epileptic encephalopathies, such as GEFS+, Doose syndrome, EIMFS, West syndrome, LGS, Rett syndrome, and NEE. In addition to epilepsy, hemiplegic migraine, ASD, sudden death, and arthrogryposis multiplex congenital can also be caused by mutations of *SCN1A*.

## Introduction

Voltage-gated sodium channel (VGSC) channels play an essential role in normal neurological function (1), especially in the initiation and propagation of action potential. To date, nine α subunits of sodium channels have been found and confirmed (Nav1.1–Nav1.9). These channels are composed of four homologous but distinct domains (DI–DIV), each of which contains six transmembrane segments (S1–S6) (2) (**Figures 2**, **3**). *SCN1A*, a Nav1.1 α subunit composed of 26 coding exons and located in the 85-kb gene region, is the most common epileptic gene and the most common pathogenic gene in the Dravet syndrome (DS), a catastrophic and intractable epileptic encephalopathy (EE) (3). Phenotypes caused by *de novo SCN1A* pathogenic variants are very variable, ranging from the severely affected patients with DS to much milder cases of genetic epilepsy febrile seizures plus (GEFS+). In addition to gene mutations of *SCN1A* that can cause DS, other genes include *PCDH19, SCN2A, SCN8A, SCN1B, GABRA1, GABRG2, GABRB3, STXBP1, HCN1, CHD2*, and *KCNA2* can also cause DS or DS-like phenotypes (4). They are also closely related to other epileptic diseases and nonepileptic diseases (5–10).

## Methods

### Literature Search

A systematic search was performed in PubMed and SCOPUS. The most recent search was performed on October 20, 2021, using the term “*SCN1A*” or “scn1a”.

### Data Extraction

**Figure 1:**
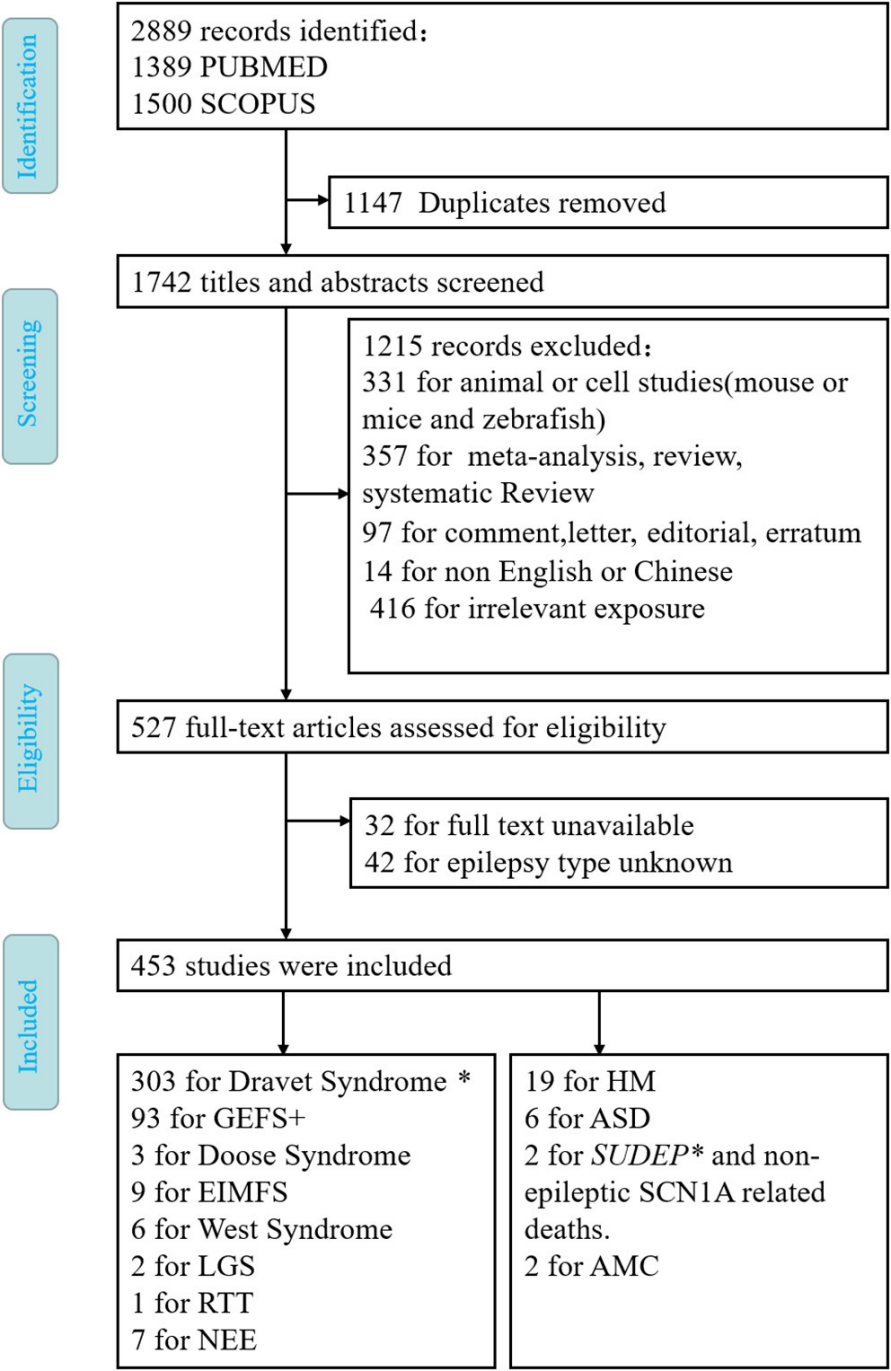
Flow diagram depicting search process and study selection. *means that SUDEP often occurs in Dravet syndrome.

All the articles with mutations of *SCN1A* associated with a particular disease were included in the criteria. We excluded articles not written in English or Chinese, nonoriginal work that has nothing to do with people, such as reviews, meta-analysis, animals or cells, experimental articles not adding information to the question posed in this review, and papers that could not be retrieved *via* PubMed or SCOPUS. The records were screened by JD and evaluated by XL with respect to the inclusion and exclusion criteria. Disagreements were resolved through a discussion between the two review authors (Figure 1).

## Results

After the elimination of duplicates (1,147 articles), the literature search yielded 1,742 articles (Figure 1). After screening all the abstracts, 1,215 records were excluded. Thus, 527 articles were included in the full-text analysis. Of these, 74 full-text articles were excluded. Articles were excluded based on the following exclusion criteria: animal or cell studies (*n* = 331); review, systematic review, and meta-analysis (*n* = 357); comment, letter, editorial, and erratum (*n* = 97); reports not in English or Chinese (*n* = 14); irrelevant exposure (*n* = 416); full text unavailable (*n* = 32); or epilepsy type unknown (*n* = 42). Finally, 453 studies met the inclusion and did not meet the exclusion criteria (Figure 1). It is well known that *SCN1A* is the main pathogenic cause of DS and GEFS+. Therefore, we only briefly describe *SCN1A* without discussing its specific mutation sites in detail.

### Summary of Findings

The *SCN1A* gene is not only associated with DS and GEFS+, but can also cause other disorders, including epilepsy diseases such as Doose syndrome, epilepsy of infancy with migrating focal seizures (EIMFS), West syndrome, Lennox–Gastaut syndrome (LGS), Rett syndrome, and nonsyndromic epileptic encephalopathy (NEE), as well as nonepileptic diseases such as hemiplegia migraine, autism spectrum disorder (ASD), sudden death (sudden unexpected death in epilepsy [SUDEP] and nonepileptic *SCN1A*-related sudden deaths), and arthrogryposis multiplex congenita (AMC).

## Discussion And Narrative Synthesis

Mutations in the voltage-gated sodium channel subunit gene *SCN1A* are identified predominantly in patients with DS, also known as severe myoclonic epilepsy of infancy (SMEI), and in the families with GEFS+. However, *SCN1A* is less common in epileptic and nonepileptic disorders other than DS and GEFS+. Herein, we focus on these rare diseases with the exception of DS and GEFS+.

### Dravet Syndrome

Dravet syndrome is a refractory and catastrophic EE that is mainly caused by haploinsufficiency due to a loss-of-function mutation in the *SCN1A* gene (1, 11). About 80% of DS is caused by mutations in the *SCN1A* gene. To date, more than 1,800 mutations have been identified in *SCN1A* (12, 13). Heat-induced epilepsy, the most common type of epilepsy in DS, is often caused by fever, vaccinations, and hot baths (14–16). With aging, the incidence of heat-induced epilepsy decreases, turning into the refractory epilepsy. Meanwhile, the cognitive dysfunction continues to aggravate and stabilize. Photosensitive epilepsy can also be observed in some patients with DS (17). In addition to the epileptic seizures, DS and other comorbidities that can be combined include ataxia, premature death, language, and motor development delay, cognitive impairment, sleep disorders, ASD, and SUDEP, which seriously affect the quality of life of the patients and pose a heavy economic burden to the family and society (18–23).

### *SCN1A*-Associated Non-dravet Syndrome Epilepsy

#### Genetic Epilepsy With Febrile Seizures Plus

**Figure 2:**
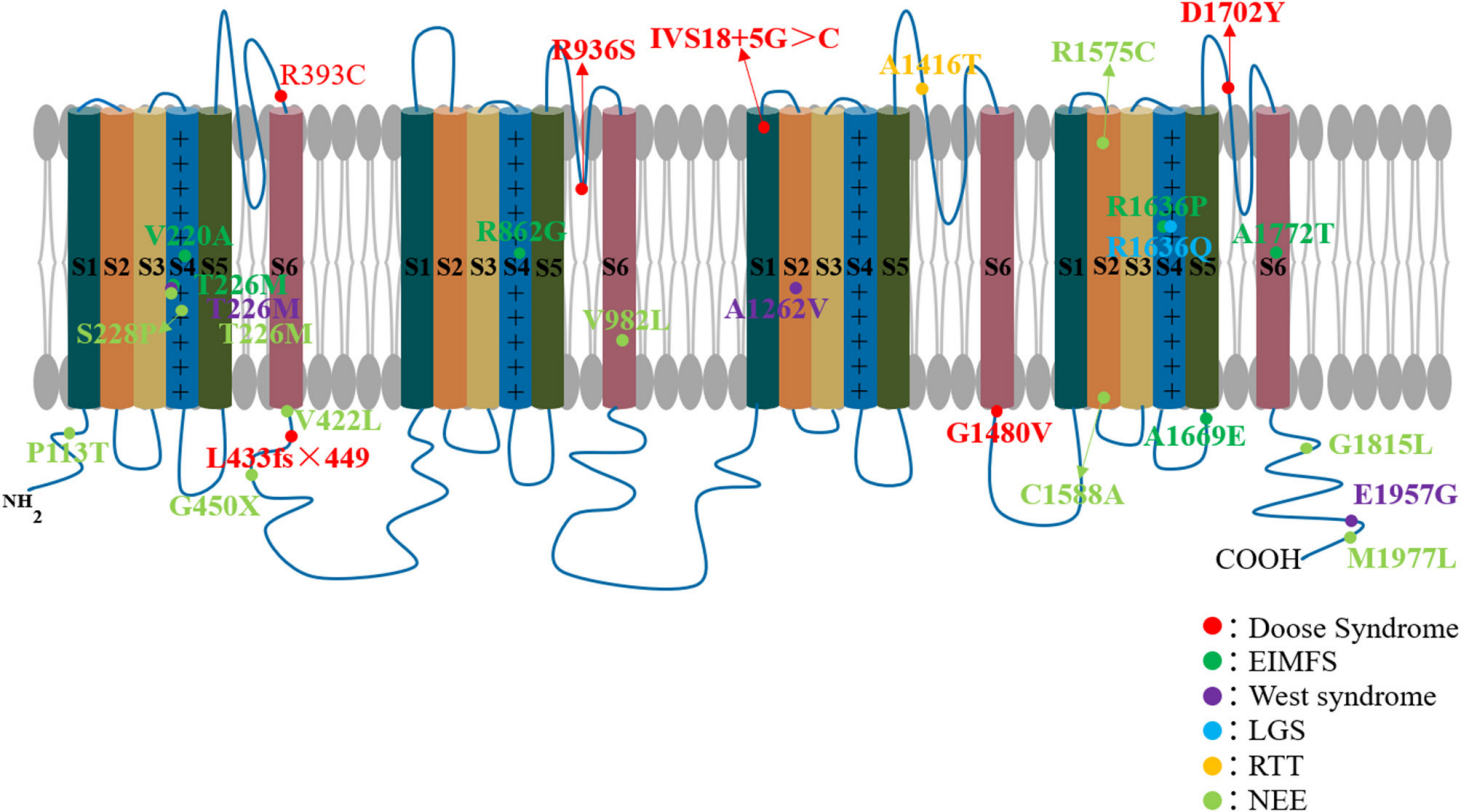
*SCN1A* mutations associated with epileptic encephalopathy. Each circular represents a patient's variant of the *SCN1A* gene.

Genetic epilepsy with febrile seizures plus, previously known as generalized epilepsy with the febrile seizures plus (FS+), was first discovered by Scheffer and Berkovic in an Australian family in 1997 (24). Since it was found that the phenotype of focal epilepsy can occur in the GEFS+ family, it was renamed genetic epilepsy with FS+. GEFS+ is an EE with a milder phenotype than DS; it is also related to the multiple gene mutations, including *SCN1A, SCN2A, SCN1B, GABRD, SCN9A, STX1B*, and *Fgf13* (25, 26). We have previously found in animal models that *GABRG2* mutations can also cause GEFS+ (27). Various clinical phenotypes can occur in the GEFS+ family, ranging from the most common febrile seizures (FS) and FS+ to the severe EE known as DS. In 2000, Escayg et al. first found mutations in the *SCN1A* gene (Thr875Met and Arg1648His) in GEFS+ families (28) (Figure 2, Table 1). Aside from DS, *SCN1A* gene mutations are the most common pathogenic genes for GEFS+. In fact, GEFS+ and DS are different manifestations of epilepsy caused by *SCN1A* mutations.

**Table 1:** Clinical data and mutation sites or chromosomal deletions in SCN1A-associated non-dravet syndrome epilepsy.

**Study**	**Toal case**	**Diseases**	***SCN1A* mutation case**	**Mutation**
Ebach et al. (29)	20	Doose syndrome	3	Frame shift: L433fs × 449; splice site variant (VS18+5G>C); (40736C>A; R946S)
Harkin et al. (30)	188	Doose syndrome	2	p.R393C; p. G1480V
Dimova et al. (31)	2	Doose syndrome	1	c.3925C>T
Angioneet al. (32)	77	Doose syndrome	1	c.5104G>T/p. D1702Y
Hinokuma et al. (33)	29	Doose syndrome	1	2q24.3, 588.7-Kb deletion
Freilich et al. (34)	1	EIMFS	1	c.C5006C>A /p. A1669E
Carranza Rojo et al. (35)	15	EIMFS	1	p.R862G; *de novo* 11.06 Mb deletion of chromosome 2q24.2q31.1
Shein et al. (36)	1	EIMFS	1	NA
Zhang et al. (37)	253	EIMFS	1	c.659T>A/ p. Val220Asp
Lim et al. (38)	5	EIMFS	3	chromosome 2q24.3 deletion
Shang et al. (39)	9	EIMFS	2	c.659T>A/pV220A; c.677G>A/p. Thr226Met
Gokben et al. (40)	35	EIMFS	1	c.4907G> C/p. R1636P
Fang et al. (41)	5	EIMFS	1	c.5314G>A/p. A1772T
Wallace et al. (42)	23	West syndrome	1	c.5870A>G/ p. E1957G
Hattori et al. (43)	1	West syndrome	1	2q24.3q31.3
Krey et al. (44)	45	West syndrome	1	c.677C>T/p.Thr226met
Na et al. (45)	150	West syndrome	1	c.3785C>T /p. Ala1262Val
Henriksen et al. (46)	2	Rett syndrome	2	g.76169G > C, c.4284 + 1G > C; g.76130G > T, c.4246 G > T/ p. Asp1416Tyr
Harkin et al. (30)	188	LGS	1	p. R1636Q
Selmer et al. (47)	22	LGS	1	c.383+1A>G
Saitoh et al. (48)	87	NEE	3	p. V982L; p.M1977L; p. R1575C
Ohashi et al. (49)#	1	NEE	1	c.1264G>T/p. Val422Leu
Mercimek-Mahmutoglu et al. (50)	110	NEE	4	c.4762T>C/p. Cys1588Arg; c.1348C>T/p. Gln450X; c337C>A/p. Pro113Thr; c.5543G>A/p. Gln1815Lys
Kobayashi et al. (51) #	11	NEE	1	c.1264G>T/p. Val422Leu
Kwong et al. (52)	26	NEE	1	splice site variant (IVS24-1G > T)
Sadleir et al. (53)	9	NEE	8	p. Thr226Met;
Spagnoli et al. (54)	1	NEE	1	c.628 T > C/p. Ser228Pro

*#This means that the two reported cases belong to the same case*.

#### Doose Syndrome

Doose syndrome, also known as epilepsy with myoclonic atonic seizure (EMAS), was previously called myoclonic astatic epilepsy (MAE), a rare childhood EE (55). First reported by Doose in 1970, the International League Against Epilepsy (ILAE) in 2010 changed its name to epilepsy with myoclonus-atonic seizures based on the characteristics of epileptic seizures (56, 57). Usually, Doose syndrome develops from seven months to 6 years, and the peak age of onset is 2 to 4 years. Children usually have normal mental and motor development before the onset. Most children start with a generalized tonic–clonic seizure (GTCS). The initial seizures can be very frequent, followed by a variety of generalized seizures, including myoclonic seizures, dystonic seizures, myoclonic–dystonic seizures, and atypical absence; a small number of children may have tonic seizures in the later stages (55).

Doose syndrome is associated with mutations in a variety of epilepsy genes, including *SCN1A*, ***SCN1B***, *CACNA1H, SLC2A1, GABRG2, CHD2, SLC6A1, STX1B, GABRB3, SYNGAP1*, and *WDR45* (33). In 2005, Ebach et al. reported three cases of EMAS with *SCN1A* gene mutations (29). In 2007, Harkin et al. found one case of Doose syndrome due to *SCN1A* mutation in 188 patients with EE (30). Interestingly, Dimova et al. also found a case of EMAS caused by *SCN1A* gene mutation in a GEFS+ family. The patient started with a febrile seizure at the age of three, after which subsequent multiple myoclonic and myoclonic–astatic seizures appeared (31). Recently, Hinokuma et al. found one microdeletion at 2q24.2 involving *SCN1A* in 29 patients with Doose syndrome (33) (Figure 2, Table 1). All of the foregoing extends the phenotype of the *SCN1A* gene mutation to Doose syndrome.

#### Epilepsy of Infancy With Migrating Focal Seizures

Epilepsy of infancy with migrating focal seizures, previously known as infantile migratory partial epilepsy (MPSI) or infantile malignant migratory partial seizure (MMPSI), is a rare and early-onset developmental EE inherited in an autosomal dominant pattern. It is characterized by onset within 6 months of birth and mainly manifests in the form of frequent, migratory, and varying types of focal seizures. Epileptic seizures are related to the multifocal EEG discharge. Similar to DS, this disease is often associated with severe cognitive impairment and motor impairment. However, unlike DS, the most common causative gene is *KCNT1* mutation.

Freilich et al. first identified the *SCN1A* mutation in a female infant diagnosed with MPSI. The female infant, who was delivered to term, developed epilepsy at 10 weeks after birth, accompanied by progressive hemiplegia, apnea, and progression of multifocal migratory partial epileptic seizures, leading to a recurrence of epileptic status and death at 9 months (34). In the same year, another case of *SCN1A* mutation was found in another patient with MPSI (35). In 2012, Shein et al. reported a case of *SCN1A* mutation-induced MPSI with good therapeutic effect assisted by hypothermia (36). In 2015, Lim et al. reported three cases with *SCN1A* mutation (MPSI) (38). In the same year, Zhang and colleagues found 46 cases of genetic mutations in 253 children with unexplained epilepsy and intellectual/developmental disabilities, of which only one was an *SCN1A* mutation causing malignant migrating partial seizures of infancy (37). In 2016, Shang et al. conducted genetic testing on nine cases of EIMFs and found that two (22.2%) carried an *SCN1A* mutation (39). Recently, Fang et al. also found one *SCN1A* mutation patient in five patients with EIMFS (41) (Figure 2, Table 1). *SCN1A* is currently considered to be the third most common type of genetic variation in EIMFS (58).

#### West Syndrome

West syndrome, also known as infantile spasms (IS), is a refractory classic EE characterized by repetitive epileptic spasms (ES) and hypsarrhythmia (44). The etiology of the West syndrome is complex and varied. Genetic studies of individuals with unexplained IS have identified over 37 genes as pathogenic (59). However, *SCN1A* was not reported in a recent review of West syndrome, indicating its rarity in this disease (42, 59). Hattori et al. reported a case of partial epileptic seizures at four months and a West syndrome infant at 8 months with characteristic facial appearance, big toe abnormalities, and developmental delay. Chromosome and gene sequencing revealed the deletion of the *SCN1A* gene and 2q31.1 region [arr 2q24.3q31.3 (166,303,447–180,982.972) × 1 (build19)] (43). In 2003, Wallace et al. found one case of *SCN1A* mutation in 23 patients with West syndrome (42). Ilona et al. found only one *SCN1A* mutation in 45 patients clinically diagnosed with West syndrome by genetic testing (44). Most recently, Na et al. performed targeted gene panel sequencing for 150 early onset DEE infants aged ≤ 3 months and only one patient with *SCN1A* mutation was found. These findings indicate that the phenotypic heterogeneity of *SCN1A* mutation has extended to West syndrome (Figure 2, Table 1).

#### Lennox–Gastaut Syndrome

*Lennox–Gastaut syndrome* is a childhood EE whose main clinical features include multiple types of drug-resistant seizures, intellectual disability, and abnormal EEG with diffuse spines slow complex wave or paroxysmal fast activity. The etiology of LGS is also complex and varied; about 75% of cases have obvious causes such as cortical malformations, posthypoxic ischemic results, postmeningitis/encephalitis, or metabolic encephalopathy, while about 25% are cryptogenic (60). LGS is associated with a variety of genetic mutations, including ion channel genes (*SCN2A, KCNT1, GABRA1, SCN8A*, and *GABRB3*), transcription regulation genes (CHD2), neurocutaneous syndrome-related genes (*TSC1* and *TSC2*), metabolic genes (*Alg13*), and others (45, 61, 62). However, *SCN1A* mutations rarely occur in LGS (30, 47). Harkin et al. found an *SCN1A* mutation in one out of 188 epileptic encephalopathy patients diagnosed with LGS (30). In 2009, Selmer and colleagues examined the *SCN1A* gene in 22 adult patients with LGS and found a mutation in one of them (47) (Figure 2, Table 1). In summary, *SCN1A* is rare, but it can still occur in LGS.

#### Rett Syndrome

Rett syndrome is a rare single-gene disease that is more prevalent in females. RTT patients usually have an early stagnation period of onset 6–18 months after birth, and then enter a rapid regression period of development. The typical phenotype includes intractable epileptic seizures and severe mental retardation, particularly a rapid regression in language and limited progress in the psychomotor development. They may also be accompanied by the related complications such as autism, hand stereotypes, and respiratory pattern disorders (63). While more than 95% of patients carry *de novo* mutation(s) in the methyl-CpG-binding protein 2 (*MECP2*) gene (classical RTT), a small fraction of the patients (atypical RTT) may carry genetic mutations in other genes, such as the cyclin-dependent kinase-like 5 (*CDKL5*) and *FOXG1* (64, 65).

The role of *SCN1A* dysfunction in RTT has also been highlighted by a few cases (46). Henriksen and colleagues (46) reported two patients with RTT caused by mutations in *SCN1A*. The first case is a 19-year-old female who developed febrile seizures at 5 months of age and subsequently developed afebrile focal seizures and intractable generalized seizures, including myotonic, tonic, and tonic–clonic. She also had several episodes with convulsive status epilepticus. She manifested normal hand functions and started to use a few words until she was 15 months old, but after that, her development slowed down. She stopped using her hands, her gait became broad and ataxic, and her speech disappeared. Between 1 and 2 years of age, she developed autism. At the age of 19, she still had dysmotility of hands and ataxia and suffered from breath holding and teeth grinding. Her height was only 141 cm. Her clinical signs and symptoms were consistent with classic RTT. Genetic testing showed that she was negative for MECP2, CDKL5, and FOXG1 genes, which are common to RTT, but *SCN1A* mutations were found. The second case occurred in a 32-year-old woman. She had her first febrile bilateral tonic–clonic seizure when she was 7 months old. The seizures worsened between the ages of one and two. Like the first patient, she grew normally until 12 to 15 months of age, but later acquired developmental disabilities and began to lose acquired skills. Her hand functions gradually declined, her speech disappeared, and she no longer seemed interested in her surroundings. She also suffered from bruxism and hand-washing stereotypes. At age of 32, she could walk for a few meters with support but still had ataxic and apraxic hand movements. She could not speak and had slight scoliosis. Epilepsy was always present. She also met the classic diagnostic criteria for RTT. Whole-exome sequencing unveiled the variant in *SCN1A* (Figure 2, Table 1).

#### Nonsyndromic Epileptic Encephalopathy

Developmental and epileptic encephalopathies (DEEs), also known as early onset epileptic encephalopathies, early infantile epileptic encephalopathies (EIEEs), or early infantile-onset developmental and epileptic encephalopathies (EIODEEs) (45, 51, 66), comprise a kind of refractory epileptic encephalopathy that is mainly characterized by early-onset in neonates or infants, refractory epileptic seizures, and severe abnormal electroencephalogram discharge, psychomotor retardation, or regression. DEEs include early myoclonic encephalopathy (EME), Otahara syndrome, EIMFS, West syndrome, and DS (57). Nonsyndromic epileptic encephalopathy (NEE) can be referred to as clinically diagnosed epileptic encephalopathy without the inclusion of a specific syndrome or epileptic disorder (51).

In 2014 and 2016, Japanese scholars Ohashi and Kobayashi et al. described a distinct *SCN1A* phenotype called early infantile *SCN1A* encephalopathy, in which the patient had an apparent movement disorder (49, 51). Sadleir et al. also reported eight cases of *SCN1A* mutation with hyperkinetic movement disorder in 2017 (53) (Figure 2, Table 1). This may become a new type of epileptic encephalopathy shortly. Similarly, *SCN1A* mutations are rarely found in other cases of NEE (48, 52, 54).

### *SCN1A*-Associated Nonepileptic Disease

#### Hemiplegic Migraine

**Figure 3:**
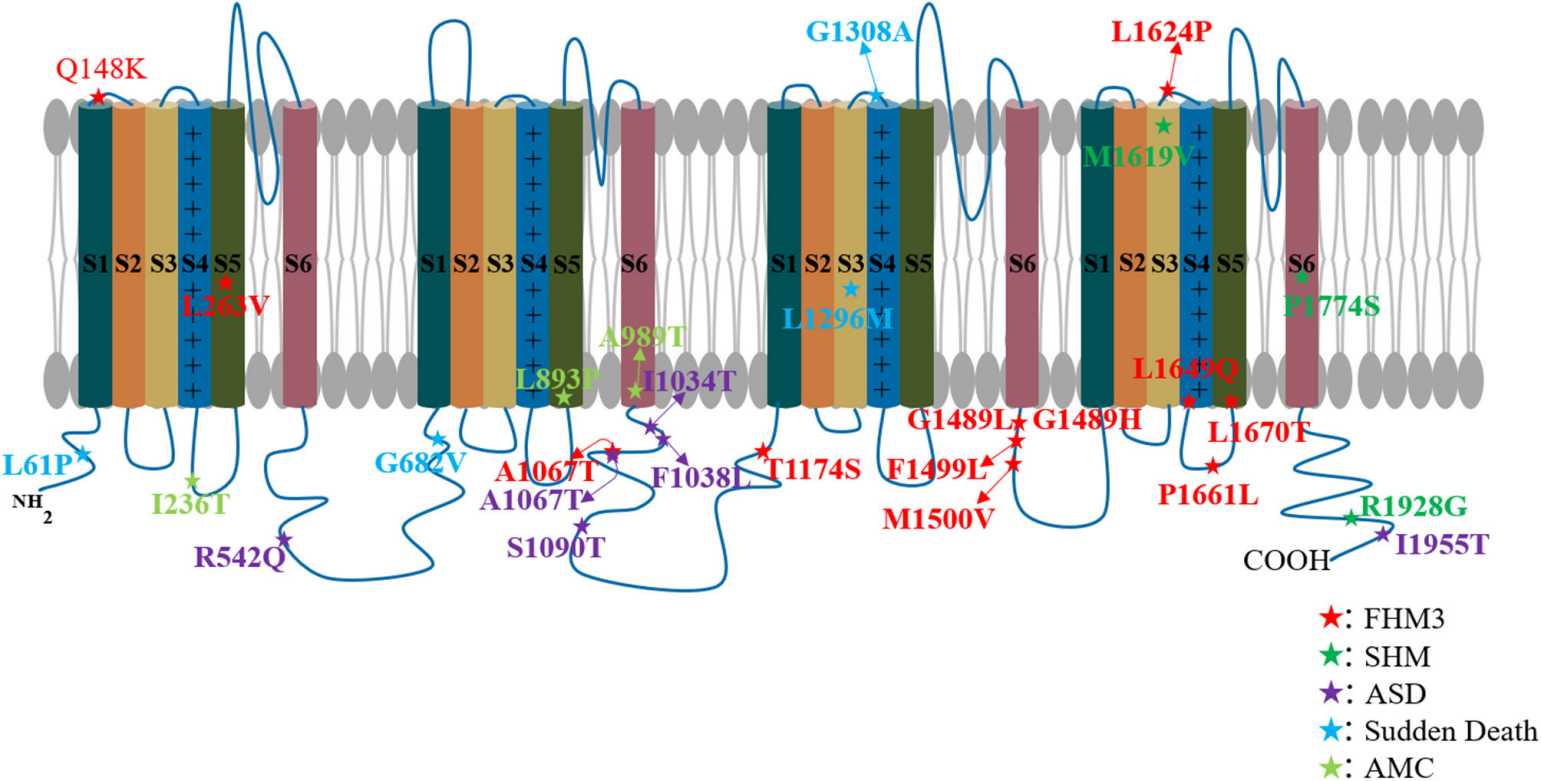
SCN1A mutations associated with non-epileptic disease. Each asterisk represents a patient's variant of the *SCN1A* gene.

Hemiplegic migraine is the most common neurological disorder that often presents with aura, which is associated with sensory and motor disturbances (67). Familial (FHM) and sporadic (SHM) hemiplegic migraines are severe subtypes of migraine associated with transient hemiparesis (68). FHM, a rare autosomal dominant genetic disorder, is a subtype of migraine with aura (MA) (69). The common classification and pathogenic genes are *CACNAIA* (FHM1), *ATP1A2* (FHM2), and *SCN1A* (FHM3) (70). Familial hemiplegic migraine type 3 (FHM3) is seldom caused by mutations in *SCN1A* (71). Martin et al. first identified the *SCN1A* mutation in 2005 in the three familial migraine families (5). Subsequently, numerous *SCN1A* mutations have been found in FMH3 and, currently, about 60 patients carry *SCN1A* mutations (67, 72–85). In addition to FHM, *SCN1A* mutations are also found in a very small number of sporadic hemiplegic migraine patients (68, 86, 87) (Figure 3, Table 2). Therefore, it is confirmed that *SCN1A* is one of the pathogenic genes for hemiplegic migraine.

**Table 2:** Clinical data and mutation sites or chromosomal deletions in SCN1A-associated non-epileptic disease.

**Study**	**Toal case**	**Diseases**	***SCN1A* mutation case**	**Mutation**	**Family**
Dichgans et al. (5)	20 families	FHM3	1	c.4465C>A/p. G1489L	European family
Gargus et al. (74)	1 family	FHM3	2	c.3521C>G/p. T1174S	Mixed European, French Canadian, Native American, and Mexican ancestry
Vanmolkot et al. (76)	10 families	FHM3	1	c.4946T>A/p. L1649Q	Netherlands family
Vahedi et al. (77)	2 families	FHM3	2	c.4495T>C/p. P1499L c.4467G>C/p. G1489H	Swiss family and French family
Castro et al. (78)#	1 family	FHM3	1	p. L263V	Portuguese family
Frosket al. (79)	1 family	FHM3	1	c.3521C>G/p. T1174S	Canada family
Zhang et al. (73)	1 family	FHM3	1	c.5009T>G/p. L1670T	Chinese Polish
Domitrz et al. (84)	60 patients	FHM3	1	p.M1500V	Polish Polish
Fan et al. (85)	1 family	FHM3	3	p. Leu1624Pro	Germany family
Weller et al. (83)	2 families	FHM3	9	p. Ile1498Met; p. Phe1661Leu	Spanish family
Barros et al. (80)	1 family	FHM3	1	p. L263V	Portuguese family
Schubert et al. (81)	2 families	FHM3	2	c.4495T>C/p. F1499L	Germany family
Khaiboullina et al. (67)	13 patients	FHM3	3	c.787C > G/p. L263V c.3521C>G/p. T1174S c.4450C>A/p. Q148K	Tatars family in Russian
Shao et al. (71)	1 family	FHM3	1	c.4495T>C	Chinese family
Pelzer et al. (82)	208 patients	FHM3	26	NA	Netherlands family
Kowalska et al. (72)	170 patients	FHM3	4	c.3199G>A/p. A1067T	Poland family
Virus et al. (68)	39 patients	SHM	1	p. R1928G	/
Chastan et al. (86)	1 patient	SHM	1	c.5321T >C/p. Phe1774Ser	/
Dube et al. (87)	1 patient	SHM	1	c.4855A>G; p. Met1619Val	/
Weiss et al. (6)	117	ASD	5	p. R542Q; p. I1034T; p. F1038L; p. A1067T; p. I1955T	/
O'Roak et al. (88)	20	ASD	1	p. P1894L	/
Koshimizu et al. (89)	28	ASD	2	c.342_344delinsAGGAGTT; c.4313T>A/p.M1438K	/
D'Gama et al. (7)	55	ASD	2	c.602+1G>A; c.4319C>T p. A1440V	/
Alvarez-Mora et al. (90)	50	ASD	1	p. R604H	/
Yin et al. (91)	134	ASD	2	c.4852 +1G > T; c.3269G > C p.Ser1090Thr	/
Matt Halvorsen et al. (92)	9	Sudden Death	1	c.182T>C/p. Leu61Pro	/
Brownstein et al. (9)	10	Sudden Death	2	c.2045G>T/ p.G682V; c.3886T>A/p. L1296M and c. 3924A>T, p. Glu1308Asp	/
Jaber et al. (93)	3	AMC	3	p. Leu893Phe; p. Ala989Thr; p. Ile236Thr	/
Laquerriere et al. (94)	315	AMC	3	NA	/

*#The patient was complicated with intractable myoclonic epilepsy*.

#### Autism Spectrum Disorder

Autism spectrum disorder is a complex psychiatric disorder characterized by impaired communication and social skills, and also restricted and repetitive behaviors (95). ASD can occur by itself or as a complication of epilepsy such as DS (6, 88, 96). DS caused by *SCN1A* gene mutation is associated with ASD (22, 96–98). Li et al. evaluated 37 patients with DS, nine of whom (24.3%) met autism criteria. They also found that people with autism had more severe intellectual disabilities than people without autism (97). Han et al. found an autism-like phenotype in *SCN1A*-mutated DS model mice (99). Interestingly, low-dose Clonazepam (a positive allosteric regulator of GABAAR) was used to mitigate this symptom, suggesting that GABAgic neurons may be directly related to ASD (99). Autism spectrum disorders can last from childhood to adulthood and even throughout life. Berkvens et al. conducted a follow-up on 13 patients with DS, among whom eight (61.5%) were classified as having ASD (96). Furthermore, ASD can occur in isolation from epilepsy. Weiss et al. found five missense mutations in patients with autism (6). Roak et al. also found one case of *SCN1A* missense mutation (p.Pro1894Leu) in 20 patients with ASD, and this mutation may be inherited from its parent (88). A recent study of 134 cases of autism identified 16 variants and 12 genes with evidence of pathogenicity, including three *SCN1A* mutations (91). In summary, *SCN1A* is closely related to ASD and has been considered as an ASD candidate gene (6, 7, 11, 89, 90) (Figure 3, Table 2).

#### Sudden Unexpected Death in Epilepsy and Non-epileptic SCN1A-Related Sudden Deaths

Epilepsy-related deaths include seizures leading to asphyxia, injury, drowning, the occurrence of epileptic status, suicide, and SUDEP, which is a common cause of death in patients with epilepsy (100). SUDEP is a sudden, accidental death of a person with epilepsy, with or without witnesses, not from trauma or drowning, and with or without epileptic seizures; an epilepsy status must be ruled out and no structural or toxic cause of death is found at autopsy (101, 102). SUDEP generally occurs in 1.2 per 1,000 people with epilepsy per year (101). The sodium channels *SCN1A, SCN1B*, and *SCN5A* are considered as genes related to SUDEP (8, 103–106). DS, which is mainly caused by the *SCN1A* mutation gene, is the best model for studying the *SCN1A* gene (107). The mortality rate in patients with DS is about 20%, with SUDEP generally present in the deaths of children and adults with epileptic status (108). SUDEP occurs at a higher rate in DS than in other childhood epilepsies, accounting for up to about 50–60% of mortality (109, 110).

Sudden death associated with *SCN1A* mutations has also been reported in nonepileptic patients. In 2016, Halvorsen et al. (92) found one *SCN1A* mutant aged 20.8 months among nine children with sudden disease who died of the unknown causes. The child developed normally with a history of febrile convulsions but, interestingly, her siblings were diagnosed with DS. In 2018, Brownstein et al. (9) found an association between *SCN1A* mutation and sudden death in younger infants. The first case is a girl who died suddenly at the age of 2 months, with the cause of death recorded as sudden infant death syndrome (SIDS). Gene sequencing revealed an *SCN1A* mutation. Microscopic examination of the hippocampus revealed focal bilamination of the dentate gyrus. The other case occurred in a 7-week-old female with two *SCN1A* mutations (92) (Figure 3, Table 2). These results suggest that *SCN1A* mutations are not only closely related to SUDEP but also associated with nonepileptic-related sudden death.

The exact mechanism of SUDEP remains unclear. In systemically knockout heterozygous *SCN1A*+*/-* mice, severe arrhythmias were found to be characterized by prolonged PR interval, increased heart rate variability, and even atrioventricular block, suggesting that changes in the cardiac *SCN1A* may be related to SUDEP (111). In another study, paroxysmal chronic bradycardia and associated ventricular electrical dysfunction were found in heterozygous *SCN1A*^+/−^ mice; notably, atropine and *N*-methyl scopolamine were effective in preventing sudden death in mice (112). In addition, respiratory dysfunction was also found in mouse models of DS, which may also be one of the causes of SUDEP in *SCN1A* mutant mice (109).

#### Arthrogryposis Multiplex Congenita

Arthrogryposis multiplex congenita refers to an etiologically heterogeneous condition that is characterized by the congenital joint contractures in two or more body areas (113). AMC is generally thought to be the downstream result of a reduction in the fetal movements. AMC has an overall incidence of one in 3,000 to 5,000 (114).

Although over 320 genes have been implicated, exemplifying the genetic heterogeneity of the condition (115), AMC is poorly related to *SCN1A*, with only two reports documented (93, 94). The first report described *SCN1A* mutations in three infants with AMC from three different families (93). During the fetal period, they are characterized by abnormal development of different joints and a lack of fetal movements (in family 1, bilateral flexion of both hands, hyperextension of knees, and reduced swallowing; in family 2, arthrogryposis of the upper limbs and microretrognathism; in family 3, bilateral camptodactyly, hyperextension of knees, and hallux valgus of feet). It is noteworthy that one of the infants (family 1) developed refractory epilepsy 2 days after birth, while the other two patients both died due to early termination of pregnancy. This suggests that in addition to peripheral joint dysplasia, AMC patients may also have abnormalities of the central nervous system, such as epilepsy, which may be similar to DS. The other description was reported by Laquerriere et al., who sequenced 315 patients with AMC and found 51 gene mutations in 166 (52.7%), including the rare *SCN1A* (94) (Figure 3, Table 2).

## Conclusion

**Figure 4:**
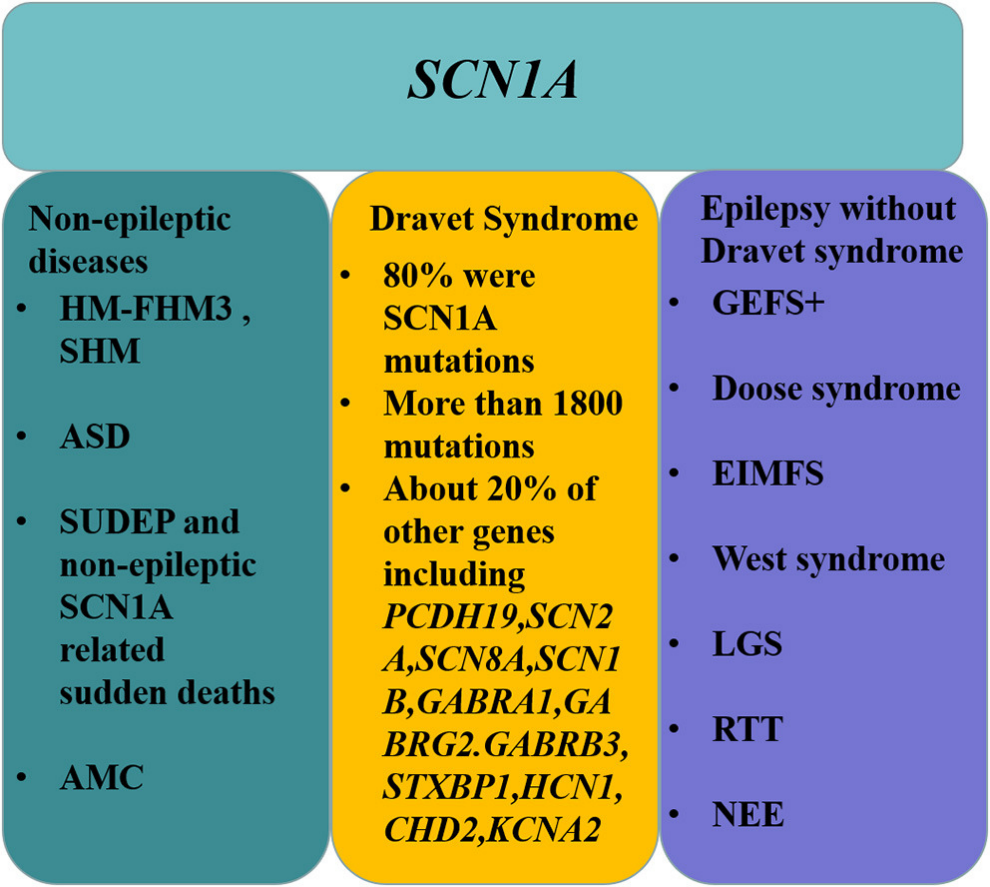
Outline of diseases associated with the *SCN1A* gene.

*SCN1A* not only causes DS and GEFS+; other epileptic encephalopathies, such as Doose syndrome, EIMFS, West syndrome, LGS, RTT, and NEE, are also directly related to *SCN1A*. In addition to epilepsy, FHM3, SHM, ASD, sudden death, and AMC can also be caused by *SCN1A* mutations (Figure 4). This review serves as a reminder to epilepsy specialists that gene sequencing is only an adjunct method for diagnosing DS. The diagnosis cannot only be made by gene sequencing but must be individualized according to the clinical manifestations of the patient to formulate a better management scheme.

## Author Contributions

All authors listed have made a substantial, direct, and intellectual contribution to the work and approved it for publication.

## Funding

This study was supported by the National Natural Science Foundation of China, Grant/Award Number: 81971085, and the Advantages Discipline Group Project of Ningxia Medical University, Grant/Award Number: XY201511.

## Conflict of Interest

The authors declare that the research was conducted in the absence of any commercial or financial relationships that could be construed as a potential conflict of interest.

## Publisher's Note

All claims expressed in this article are solely those of the authors and do not necessarily represent those of their affiliated organizations, or those of the publisher, the editors and the reviewers. Any product that may be evaluated in this article, or claim that may be made by its manufacturer, is not guaranteed or endorsed by the publisher.
